# Real experience of young and middle-aged hemodialysis patients: a qualitative meta-synthesis

**DOI:** 10.3389/fmed.2025.1530465

**Published:** 2025-04-07

**Authors:** Kai Zhang, Zeng Teng, Ailing Li, Nan Zhang, Ruixue Wang, Shuimiao Wei, Cuiping Ni

**Affiliations:** ^1^School of Nursing, China Medical University, Shenyang, Liaoning, China; ^2^Affiliated Hospital of Qingdao University, Qingdao, Shandong, China; ^3^Shenyang Orthopedics Hospital, Shenyang, Liaoning, China

**Keywords:** hemodialysis, qualitative research, meta synthesis, nursing, young and middle aged

## Abstract

**Background:**

About 89% of global End-stage renal disease (ESRD) patients receive hemodialysis. Data show that about 40% ~ 60% of dialysis patients are young and middle-aged. Hemodialysis significantly impacts the daily life and rehabilitation of patients, underscoring the urgency of understanding their experiences and needs. However, findings from previous individual qualitative studies may lack representativeness.

**Aims:**

This study used Meta-synthesis to offer a thorough understanding of the lived experiences, psychological states, and needs of young and middle-aged hemodialysis patients.

**Design:**

Systematic review and meta-synthesis.

**Methods:**

A systematic search was conducted in PubMed, Embase, The Cochrane Library, Web of Science, CINAHL, CBM, CNKI, WanFang, and VIP databases from the establishment of each database to November 4, 2024, targeting qualitative studies on the experiences of young and middle-aged hemodialysis patients (aged 18–65 years old). Quality assessment used the Joanna Briggs Institute’s 2016 Checklist for Qualitative Research, followed by meta-synthesis. The study’s reporting was informed by the principles of the Enhancing Transparency in Reporting the Synthesis of Qualitative Research (ENTREQ) framework. The thematic analysis approach was employed to synthesize the findings.

**Results:**

Twenty-two studies were included, covering 14 countries. The inclusion of 22 studies yielded 83 findings, categorized into nine subthemes and condensed into four overarching themes: negative emotional experiences toward illness and hemodialysis, experience of financial problems due to long-term hemodialysis treatment, disruptions to normal life, and seeking multiple forms of support among hemodialysis patients.

**Conclusion:**

Healthcare providers should attach importance to this group, to meet their specific needs, and support their active recovery. The goal is to help them relieve negative emotions, reduce financial burden, return to normal life and meet multiple forms of support.

## 1 Introduction

Hemodialysis (HD) is the renal replacement therapy in patients with acute and chronic renal failure, and is one of the most commonly used blood purification methods. It utilizes hemodialysis to remove metabolites and toxic substances from the human body and to correct water, electrolyte and acid–base balance disorders (1). Most hemodialysis patients undergo regular long-term dialysis sessions lasting 3.5–4.5 h, three times a week. Globally, approximately 89% of end-stage renal disease (ESRD) patients rely on hemodialysis (2). Data from 2013 indicated that 1 million people were receiving hemodialysis treatment worldwide, and the total number of people receiving dialysis was increasing by 10% per year (3). By 2023, China’s hemodialysis patient population had reached 910,000, and the number continues to increase significantly (4). About 40~60% of dialysis patients are young and middle-aged adults (5).

With advancements in hemodialysis technology, the survival rate of ESRD patients has markedly improved. However, prolonged treatment exerts comprehensive impacts on patients’ physical and mental health, social integration, daily functioning, and financial stability, significantly reducing quality of life (6, 7). While hemodialysis alleviates symptoms and prolongs survival, young and middle-aged patients face intensified challenges, including complications, economic strain, and treatment-related lifestyle constraints, which disproportionately burden those navigating pivotal life stages—pursuing education, advancing careers, and establishing families (8). Struggling to reconcile health demands with societal and familial obligations, they endure the dual pressures of maintaining survival and pursuing life aspirations (8). Understanding the impact of hemodialysis on young and middle-aged patients enables healthcare providers to deliver targeted nursing care and support.

Meta-synthesis is a method of systematic integration of qualitative research results, aiming to form a more comprehensive and in-depth understanding of a phenomenon by extracting common themes and deep meanings from different studies. Unlike the Meta-analysis of traditional quantitative research, Meta-synthesis focuses on the human experience, emotion, and socio-cultural context contained in qualitative data, and is particularly suitable for exploring complex health issues (such as psychological adaptation processes in patients with chronic diseases). There are many qualitative studies on the experience and needs of young and middle-aged hemodialysis patients worldwide, but the sample size of a single study is small, and it is difficult to fully reflect the real situation of the target population by cultural background or sample characteristics. Meta-synthesis can integrate the results of different studies, reveal common rules, and provide more universal evidence for clinical practice. This study used Meta-synthesis to offer a thorough understanding of the lived experiences, psychological states, and needs of young and middle-aged hemodialysis patients.

## 2 Methods

### 2.1 Research design

The Joanna Briggs Institute’s (JBI) PICo framework, an Australian evidence-based model (9), informed the development of our inclusion and exclusion criteria, using “P” for the study population; “I” for the research focus, the phenomenon of interest; and “Co” for the research context. The study’s reporting was informed by the principles of the Enhancing Transparency in Reporting the Synthesis of Qualitative Research (ENTREQ) framework (10) (Supplementary material). The thematic analysis approach was employed to synthesize the findings (11).

### 2.2 Literature search strategy

Zhang Kai and Teng Zeng searched PubMed, Embase, The Cochrane Library, Web of Science, CINAHL, CNKI, CBM, Wanfang, and VIP databases. The search integrated subject terms and free terms, customized for each database’s unique characteristics. Search terms included “hemodialysis,” “MHD,” “hemodialysis,” “renal dialysis,” “young people,” “young*,” “adult*,” “middle-aged adult,” “experience*,” “feel*,” “opinion,” “need*,” “demand*,” “perception*,” “attitude*,” “qualitative research,” “qualitative study,” “grounded theory,” “phenomenological analysis,” “interview*,” “focus group,” and “ethnography.” We conducted a manual search using the literature tracing method to comprehensively supplement the literature. Our search encompassed the period from the establishment of each database to November 4, 2024. The study is registered in Prospero: CRD42024577165. Table 1 illustrates the Search strategy.

**Table 1:** Search strategy.

Literature library	Search queries	Search results
WanFang	主题:(血透 OR 血液透析 OR 维持性血液透析 OR MHD)AND(中青年 OR 中年 OR 青年 OR 年轻)AND(体验 OR 感受 OR 经历 OR 经验 OR 感觉)AND(质性研究 OR 现象学研究 OR 扎根理论 OR 人种志 OR 定性研究 OR 民族志)	140
CNKI	主题:(血透 + 血液透析 + 维持性血液透析 + MHD)AND(中青年 + 中年 + 青年 + 年轻) AND (体验 + 感受 + 经历 + 经验 + 感觉)AND(质性研究 + 现象学研究 + 扎根理论 + 人种志 + 定性研究 + 民族志)	2
VIP	主题:(血透 OR 血液透析 OR 维持性血液透析 OR MHD)AND(中青年 OR 中年 OR 青年 OR 年轻)AND(体验 OR 感受 OR 经历 OR 经验 OR 感觉)AND(质性研究 OR 现象学研究 OR 扎根理论 OR 人种志 OR 定性研究 OR 民族志)	16
CBM	(“血透”[常用字段:智能] OR “血液透析”[常用字段:智能] OR “维持性血液透析”[常用字段:智能] OR “MHD”[常用字段:智能]) AND(“中青年”[常用字段:智能] OR “中年”[常用字段:智能] OR “青年”[常用字段:智能] OR “年轻”[常用字段:智能]) AND(“体验”[常用字段:智能] OR “感受”[常用字段:智能] OR “经历”[常用字段:智能] OR “经验”[常用字段:智能] OR “感觉”[常用字段:智能]) AND(“质性研究”[常用字段:智能] OR “现象学研究”[常用字段:智能] OR “扎根理论”[常用字段:智能] OR “人种志”[常用字段:智能] OR “定性研究”[常用字段:智能] OR “民族志”[常用字段:智能])	8
Pubmed	#1(((“young people”[Title/Abstract]) OR (young*[Title/Abstract])) OR (adult*[Title/Abstract])) OR (“middle-aged adult”[Title/Abstract])	2,342,053
	#2 ((((((hemodialysis[Title/Abstract]) OR (hemodialysis[MeSH Terms])) OR (hemodialysis[MeSH Terms])) OR (hemodialysis[Title/Abstract])) OR (MHD[Title/Abstract])) OR (“renal dialysis”[Title/Abstract])) OR (“renal dialysis”[MeSH Terms])	159,795
	#3((((((experience*[Title/Abstract])OR (feel*[Title/Abstract])) OR (opinion[Title/Abstract])) OR (need*[Title/Abstract])) OR (demand*[Title/Abstract])) OR (perception*[Title/Abstract])) OR (attitude*[Title/Abstract])	4,504,314
	#4(((((((((“qualitative research”[Title/Abstract]) OR (“qualitative research”[MeSH Terms])) OR (“qualitative study”[Title/Abstract])) OR (“grounded theory”[Title/Abstract])) OR (“grounded theory”[MeSH Terms])) OR (“phenomenological analysis”[Title/Abstract])) OR (interview*[Title/Abstract])) OR (“focus group”[Title/Abstract])) OR (ethnography[MeSH Terms])) OR (ethnography[Title/Abstract])	734,039
	#5 = #1 AND #2 AND #3 AND #4	226
Web of science	#1(((TS = (“young people”)) OR TS = (young*)) OR TS = (adult*)) OR TS = (“middle-aged adult”) and Preprint Citation Index (Exclude – Database)	9,739,347
	#2(((TS = (hemodialysis)) OR TS = (hemodialysis)) OR TS = (MHD)) OR TS = (“renal dialysis”) and Preprint Citation Index (Exclude – Database)	239,826
	#3((((((TS = (experience*)) OR TS = (feel*)) OR TS = (opinion)) OR TS = (need*)) OR TS = (demand*)) OR TS = (perception*)) OR TS = (attitude*) and Preprint Citation Index (Exclude – Database)	10,995,258
	#4((((((TS = (“qualitative research”)) OR TS = (“qualitative study”)) OR TS = (“grounded theory”)) OR TS = (“phenomenological analysis”)) OR TS = (interview*)) OR TS = (“focus group”)) OR TS = (ethnography) and Preprint Citation Index (Exclude – Database)	1,463,016
	#5 = #1 AND #2 AND #3 AND #4	871
Cochrane library	#1(middle-aged adult): ti,ab,kw OR (adult*): ti,ab,kw OR (young*): ti,ab,kw OR (young people): ti,ab,kw	928,314
	#2(hemodialysis): ti,ab,kw OR (hemodialysis): ti,ab,kw OR (MHD): ti,ab,kw OR (renal dialysis): ti,ab,kw	20,611
	#3(experience*): ti,ab,kw OR (feel*): ti,ab,kw OR (opinion): ti,ab,kw OR (need*): ti,ab,kw OR (demand*): ti,ab,kw OR (perception*): ti,ab,kw OR (attitude*): ti,ab,kw	426,322
	#4(qualitative research): ti,ab,kw OR (qualitative study): ti,ab,kw OR (grounded theory): ti,ab,kw OR (phenomenological analysis): ti,ab,kw OR (interview*): ti,ab,kw OR (focus group): ti,ab,kw OR (ethnography): ti,ab,kw	80,950
	#5 = #1 AND #2 AND #3 AND #4	143
Embase	#1‘young people’:ti,ab,kw OR ‘young*’:ti,ab,kw OR ‘adult*’:ti,ab,kw OR ‘middle-aged adult’:ti,ab,kw	687,785
	#2‘hemodialysis’:ti,ab,kw OR ‘hemodialysis’:ti,ab,kw OR ‘MHD’:ti,ab,kw OR ‘renal dialysis’:ti,ab,kw	148,176
	#3‘experience*’:ti,ab,kw OR ‘feel*’:ti,ab,kw OR ‘opinion’:ti,ab,kw OR ‘need*’:ti,ab,kw OR ‘demand*’:ti,ab,kw OR ‘perception*’:ti,ab,kw OR ‘attitude*’:ti,ab,kw	6,126,911
	#4‘qualitative research’:ti,ab,kw OR ‘qualitative study’:ti,ab,kw OR ‘grounded theory’:ti,ab,kw OR ‘phenomenological analysis’:ti,ab,kw OR ‘interview*’:ti,ab,kw OR ‘focus group’:ti,ab,kw OR ‘ethnography’:ti,ab,kw	3,146,628
	#5 = #1 AND #2 AND #3 AND #4	228
CINAHL	#1AB young people OR AB young* OR AB adult* OR AB middle-aged adult	4,693,676
	#2AB hemodialysis OR AB hemodialysis OR AB MHD OR AB renal dialysis	137,287
	#3AB experience* OR AB feel* OR AB opinion OR AB need* OR AB demand* OR AB perception* OR AB attitude*	14,542,996
	#4AB qualitative research OR AB qualitative study OR AB grounded theory OR AB phenomenological analysis OR AB interview* OR AB focus group OR AB ethnography	2,296,549
	#5AB (S1 AND S2 AND S3 AND S4)	220

### 2.3 Inclusion criteria

① Participants (P): Hemodialysis patients aged 18–65 were classified as young and middle-aged. ② Interest of Phenomena (I): Psychological states, needs, experiences, and emotions of hemodialysis patients aged 18–65. ③ Context (Co): Interviews with hemodialysis patients during hospital or home. ④ Study types (S): Qualitative studies using phenomenology, grounded theory, and ethnography, encompassing interviews and thematic analysis.

### 2.4 Exclusion criteria

① Exclude conference papers, policy documents, review articles, and case reports. ② Exclude articles that are published more than once or lack accessible full texts. ③ Exclude studies with a methodological quality rating of C. ④ Exclude literature that is not written in Chinese or English.

### 2.5 Literature quality assessment

Two researchers independently evaluated the included studies based on the 2016 Joanna Briggs Institute’s quality assessment criteria for qualitative research (12). They rated 10 criteria, responding “yes,” “no,” or “unclear” for each. When all 10 items are answered “yes,” the likelihood of bias is minimal and rated as A. If some of the quality criteria are met but not all, the possibility of bias is considered moderate, designated as B. If all items are answered “no,” the possibility of bias is deemed high and classified as C. Discrepancies in evaluations were resolved through discussion; a third party arbitrated if consensus was not reached. Studies graded A and B were included in the final analysis.

### 2.6 Data screening and extraction

Two researchers, both postgraduate students who have completed a course in evidence-based nursing and are familiar with qualitative methods, independently screened the literature, extracted data, and cross-verified their findings. A third researcher (NCP) mediated evaluations in cases of disagreement. The screening process included importing documents into NoteExpress for de-duplicating, removing, reviewing titles and abstracts based on established criteria, and conducting a full-text review for secondary screening. Data extraction focused on author, country, methodology, study object, the phenomenon of interest, contextual factors, and key findings.

### 2.7 Meta synthesis

The information from the included studies were integrated utilizing the three-stage thematic analysis framework proposed by Thomas and Harden (11). This process encompassed three distinct phases. Initially, a dual-review methodology was applied by ZK and NCP, who meticulously scrutinized the primary literature, engaging in repeat readings to assign codes based on the semantic and contextual nuances of the text. Proceeding to the second phase, ZK and NCP conducted a comparative analysis of the emergent codes, identifying commonalities and disparities, which facilitated the formation of descriptive themes. In the third phase, the reviewers ZK and NCP conducted a thorough review of these descriptive themes, discerning novel insights, interpretations, or presuppositions that enriched the thematic landscape.

## 3 Results

### 3.1 Literature screening process and results

**Figure 1:**
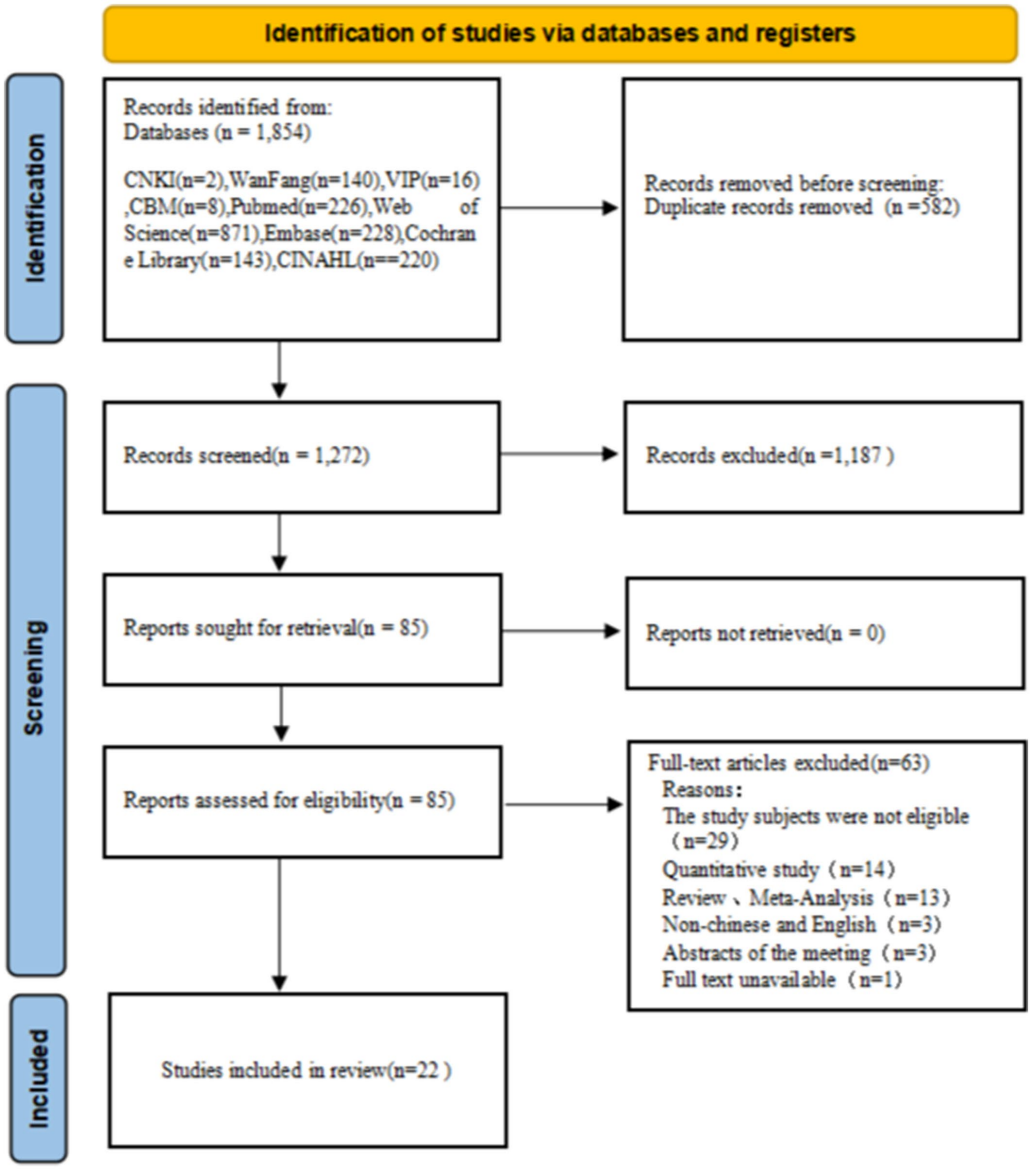
PRISMA flow diagram for article selection.

Initially, our search strategy identified 1,854 relevant articles, which was reduced to 1,272 after the removal of duplicates. Following the review of titles and abstracts, the articles were narrowed down to 85 according to the selection. The full-text review led to the exclusion of an additional 63 articles. A total of 22 articles were included after quality evaluation. The process of literature screening is depicted in Figure 1.

### 3.2 Characteristics results of the included literature

The research methods include: 16 phenomenological studies, 2 grounded theory studies, 2 descriptive qualitative studies, 1 exploratory qualitative study and 1 narrative qualitative study. Age range from 18 to 65 years old. The study involving the authentic experience of hemodialysis in young and middle-aged adults in 14 countries. Studies were conducted in the United States (*n* = 1), China (*n* = 5), Ghana (*n* = 1), Ethiopia (*n* = 2), Iran (*n* = 2), South Africa (*n* = 1), Norway (*n* = 1), The United Kingdom (*n* = 1), New Zealand (*n* = 1), Pakistan (*n* = 1), Ireland (*n* = 1), Thailand (*n* = 1), Brazil (*n* = 3), Singapore (*n* = 1). More than 50% of the articles in this paper were published less than 5 years ago, and only 2 articles were published more than 15 years ago. The basic characteristics of the included literatures are shown in Table 2.

**Table 2:** Basic characteristics of the included studies.

Author	Country	Research method	Research object(male/female)	The phenomenon of interest	Situational factor (interview venue)	Principal findings
Tang (23)	China	Penomenology(Face-to-face in-depth interviews)	20 HD patients aged 18–60 years	To understand the living experience of HD patients	Blood purification center	3 themes: 1. Emotional experience after illness 2. Heavy economic burden 3. Family role and changes in social interaction
He et al. (20)	China	Phenomenology(Face-to-face semi-structured interview)	12 HD patients aged 35–48 years (8/4)	To understand the stressors of HD patients after illness	Blood purification center	4 themes: 1. Disease aspect 2. Long-term dialysis 3. Self-concept change 4. Economic pressure
Li et al. (13)	China	Phenomenology(Face-to-face semi-structured interview)	13 HD patients aged 18–59 years (8/5)	To understand the psychological experience of HD patients during treatment	Dialysis center Outpatient clinic	4 themes: 1. Negative emotional distress 2. Positive emotional experience 3. Insufficient self-management 4. Desire to return to society
Han and Peng (24)	China	Phenomenology(Face-to-face semi-structured interview)	15 HD patients aged 18–40 years	To understand the psychological experience of young patients with HD	Dialysis room	6 themes: 1. Feeling unlucky 2. Wanting to know more about HD 3. Anxiety and depression were aggravated 4. Self-reproach 5. Unharmonious sexual life 6. Needing psychological support
Boateng et al. (16)	Ghana	Phenomenology(Face-to-face semi-structured interview)	12 HD patients aged 30–60 years (9/3)	To understand the life experience of HD patients	Dialysis room	4 themes: 1. Lacking relevant knowledge 2. Refusal of treatment 3. Financial burden 4. Lacking insurance/government subsidies
Gebrie et al. (21)	Ethiopia	Descriptive study(Semi-structured interview)	15 HD patients aged 19–63 years (9/6)	To understand the experience of HD patients after illness	A place convenient to the participant (health care facility, home, hospital)	5 themes: 1. Gratitude 2. Supportive environment 3. Aspiring for a kidney transplant 4. Restricted living 5. Invasive procedure
Rambod et al. (17)	Iran	Phenomenology(Semi-structured interviews, observations, and field notes)	14 HD patients aged 23–54 years (8/6)	To understand the hope experience of HD patients after illness	Hemodialysis center	5 themes: 1. Hoping for the future 2. Opportunities and challenges in fostering hope 3. Negative emotions 4. Coping strategies 5. Growth and excellence were the results of holding onto hope
Faria-Schützer et al. (29)	Brazil	Phenomenology(Semi-structured interviews)	11 female patients 23 to 63 with HD	To understand the experiences of women with HD during pregnancy and postpartum	Hemodialysis clinics	3 themes: 1. The relationship between kidney disease and pregnancy 2. Lacking knowledge about dialysis 3. Views after receiving dialysis
Mokwena et al. (25)	South Africa	Interpretative phenomenological analysis(Face-to-face interview)	10 HD patients aged 20–59 years	To understand the voices of black HD patients in South Africa	Home and work	3 themes: 1. Distress and fear of death after ESRD diagnosis 2. Dialysis dependence interfering with life management and sexual relationship with wife 3. Dialysis is a challenging but lifesaving procedure
Tadesse et al. (18)	Ethiopia	Phenomenology(Semi-structured interview)	12 HD patients aged 24–50 years (10/2)	To understand the living experience of HD patients	A separate room in the Hospital	6 themes: 1. The severity of the disease 2. The challenges of accepting HD 3. The financial burden 4. Limitations of life 5. Feelings of dependence 6. Psychological effects
Andersen-Hollekim et al. (27)	Norway	Narrative method(Face-to-face interview)	11 patients with HD aged 35–64 years	Learn about the dialysis experience of HD patients of working age	Hospital conference room/home/workplace	3 themes: 1. Informed, but not involved in, treatment choice 2. The patients’ work and HD treatment limited their autonomy 3. Patient trust was compromised by communication deficits
Maria (8)	The United Kingdom	Interpretative phenomenological analysis(Semi structuredinterview)	10 HD patients aged 28–49 years (4/6)	Parents receiving HD treatment, patient role experience	Consulting Room	2 themes: 1. Experience of HD treatment 2. Life experience of parents with end-stage renal disease
Walker et al. (30)	New Zealand	Phenomenology(Semi-structured interview)	25 HD patients aged 31–65 years (14/11)	To understand the patient experience of community families receiving HD	Communities and families	4 themes: 1. Reducing family burden 2. Providing flexibility and freedom 3. Taking control of their own health 4. Community Support
Ghaffari et al. (46)	Iran	Grounded theory(Semi-structured interview)	22 cases of HD aged 36–63 years (14/8)	Coping styles for stress in patients receiving HD	Dialysis center	3 themes: 1. Struggling coping 2. Meaning-based coping 3. Moderating factors
Shouket et al. (22)	Pakistan	Interpretative phenomenological analysis(Semi-structured interview)	24 patients with HD aged 21–60 years (19/5)	To understand the life experience of patients undergoing HD	Dialysis center	1 theme and 6 sub-themes: HD experience (impact of dialysis on daily life, social problems, cognitive aspects, emotional difficulties, financial difficulties, professional challenges)
Calvey and Mee (47)	Ireland	Phenomenology(Semi-structured interview)	7 HD patients aged 29–60 years	To understand the life experience of HD patients after leaving the dialysis room	The isolation room at the dialysis center	4 themes: 1. Future self 2. Living self 3. Mortal/vulnerable self 4. Learning self
Joshua (28)	United States	Exploratory qualitative study(Face-to-face semi-structured interview)	16 HD patients aged 19–30 years (10/6)	Understand the resources used and obstacles encountered in the life of patients undergoing HD	Private location (coffee shop, library, private office)	7 themes: 1. Stress and Anxiety 2. Young patients using friends, family, and prayer to cope with the illness 3. Effects of dialysis on school and employment 4. Pain relief 5 Major barriers 6. Support groups and social support 7 Recommendations for other young patients
Ko et al. (19)	China	Phenomenology(semi-structured interviews)	14 HD patients aged 36–58 years (6/8)	To explore the experience of quality of life in young and middle-aged patients with HD	Dialysis center	5 themes: 1. Physical function 2. Psychological function 3. Social function 4. Economic status 5. Overall quality of life
Yodchai et al. (48)	Thailand	Grounded theory(Semi-structured interview)	4 HD patients aged 24–64 years (3/1)	Understanding how HD patients adapt to dialysis treatment	Home	4 coping processes: 1. Planning 2. Adjusting and avoiding 3. Believing in religion and superstition 4. Living with hope
de Carvalho Pinto Nazario and Turato (26)	Brazil	Phenomenology (Semi-structured interview)	9 fertile adult patients with HD	To understand the fantasies about pregnancy and motherhood in fertile HD patients	Hospital	2 themes: 1. Quality of life, normal/abnormal, stigma, and pregnancy in HD 2. The idea of adoption as a possibility to play the role of mother
Lai et al. (14)	Singapore	Interpretative phenomenological analysis(Semi-structured interview)	13 patients aged 39–63 years with initial HD (6/7)	Understand the experiences and needs of patients starting HD	-	3 themes: 1. Emotional distress 2. Treatment-related problems 3. Social support
Campos et al. (15)	Brazil	Descriptive qualitative study(Semi-structured interview)	23 HD patients aged 41–50 years (7/16)	To understand the social representations of HD patients	Hospital	2 themes: 1. Limited knowledge of kidney diseases and 2. Visible phenomena in kidney disease

“-”: no report; “HD”, hemodialysis.

### 3.3 Quality appraisal

Twenty-one studies were rated as “B” and 1 was rated as “A,” and thus none was deleted after quality appraisal. Twenty-one studies rated “no” on item 6 “introducing researchers from a cultural or theoretical background.” 19 studies were rated “no” in item 7 “study describes the researchers’ influence on the study and vice versa.” On the other items, all 22 studies were rated as “yes.” The results of literature quality evaluation is shown in Table 3.

**Table 3:** Results of methodological quality assessment of the included studies.

Included literature	①	②	③	④	⑤	⑥	⑦	⑧	⑨	⑩	Grade of quality
Tang (23)	Yes	Yes	Yes	Yes	Yes	NO	No	Yes	Yes	Yes	B
He et al. (20)	Yes	Yes	Yes	Yes	Yes	No	No	Yes	Yes	Yes	B
Li et al. (13)	Yes	Yes	Yes	Yes	Yes	No	No	Yes	Yes	Yes	B
Han and Peng (24)	Yes	Yes	Yes	Yes	Yes	No	No	Yes	Yes	Yes	B
Boateng et al. (16)	Yes	Yes	Yes	Yes	Yes	No	No	Yes	Yes	Yes	B
Gebrie et al. (21)	Yes	Yes	Yes	Yes	Yes	No	No	Yes	Yes	Yes	B
Rambod et al. (17)	Yes	Yes	Yes	Yes	Yes	No	No	Yes	Yes	Yes	B
Faria-Schützer et al. (29)	Yes	Yes	Yes	Yes	Yes	No	No	Yes	Yes	Yes	B
Mokwena et al. (25)	Yes	Yes	Yes	Yes	Yes	No	No	Yes	Yes	Yes	B
Tadesse et al. (18)	Yes	Yes	Yes	Yes	Yes	No	Yes	Yes	Yes	Yes	B
Andersen-Hollekim et al. (27)	Yes	Yes	Yes	Yes	Yes	No	No	Yes	Yes	Yes	B
Maria (8)	Yes	Yes	Yes	Yes	Yes	Yes	Yes	Yes	Yes	Yes	A
Walker et al. (30)	Yes	Yes	Yes	Yes	Yes	No	No	Yes	Yes	Yes	B
Ghaffari et al. (46)	Yes	Yes	Yes	Yes	Yes	No	No	Yes	Yes	Yes	B
Shouket et al. (22)	Yes	Yes	Yes	Yes	Yes	No	Yes	Yes	Yes	Yes	B
Calvey and Mee (47)	Yes	Yes	Yes	Yes	Yes	No	No	Yes	Yes	Yes	B
Joshua (28)	Yes	Yes	Yes	Yes	Yes	No	No	Yes	Yes	Yes	B
Ko et al. (19)	Yes	Yes	Yes	Yes	Yes	No	No	Yes	Yes	Yes	B
Yodchai et al. (48)	Yes	Yes	Yes	Yes	Yes	No	No	Yes	Yes	Yes	B
de Carvalho Pinto Nazario and Turato (26)	Yes	Yes	Yes	Yes	Yes	No	No	Yes	Yes	Yes	B
Lai et al. (14)	Yes	Yes	Yes	Yes	Yes	No	No	Yes	Yes	Yes	B
Campos et al. (15)	Yes	Yes	Yes	Yes	Yes	No	No	Yes	Yes	Yes	B

2016 Joanna Briggs Institute’s quality assessment criteria for qualitative research. ① If the philosophical underpinnings align with the methodology; ② If the methodology matches the research question or objective; ③ If the data collection methods are consistent with the methodology; ④ If the methodology is consistent with data representativeness and analysis; ⑤ If the methodology aligns with the interpretation of results; ⑥ If the researcher’s position is explained considering cultural background and values; ⑦ If the study describes the researchers’ influence on the study and vice versa; ⑧ If the study is representative and if subjects’ views are adequately portrayed; ⑨ If the study adheres to current ethical standards; ⑩ If the conclusion is derived from data analysis and interpretation.

### 3.4 Meta-synthesis results

**Figure 2:**
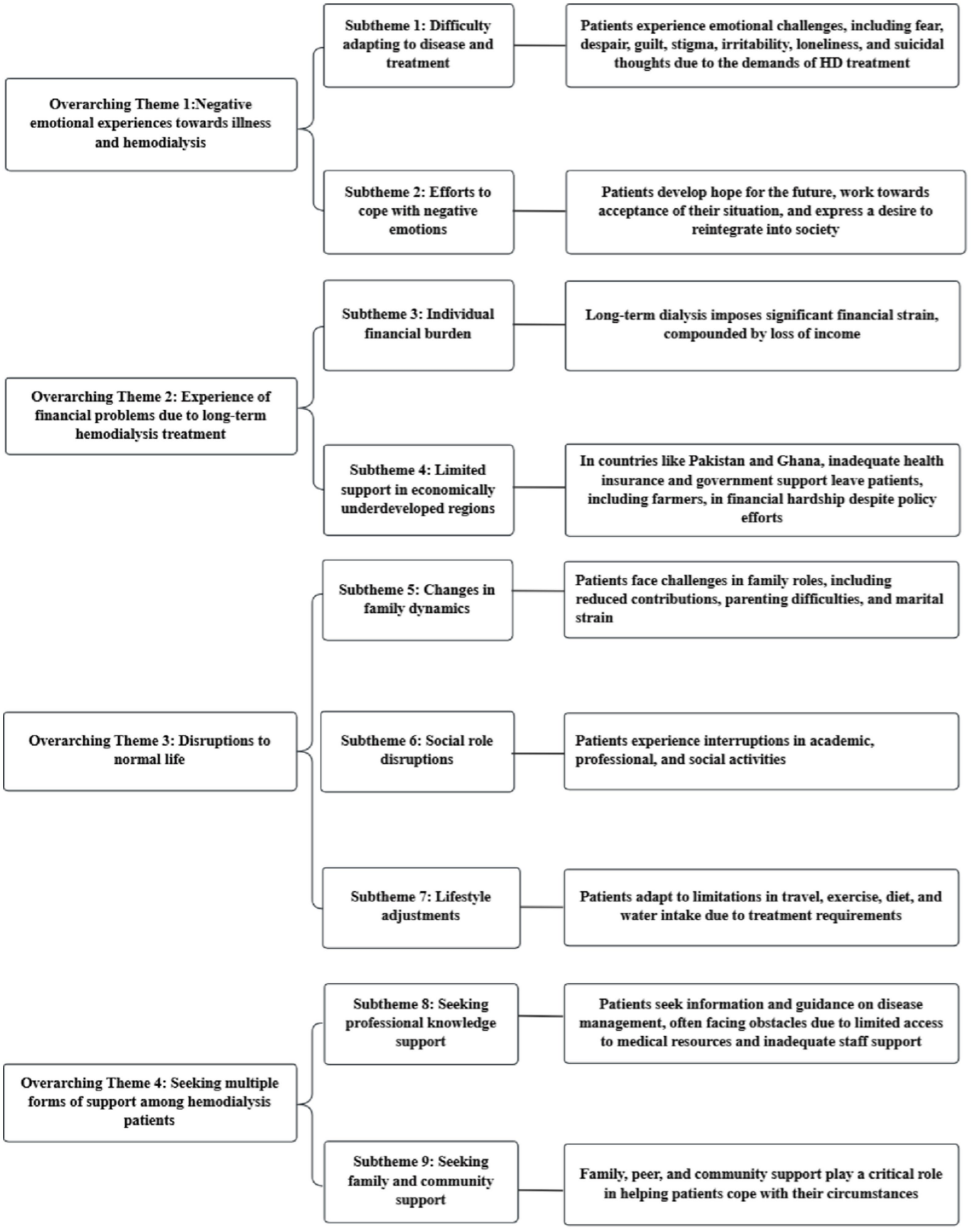
Overarching themes of included literatures. The content in the far right box is an explanation of the subtheme.

We extracted 83 themes from 22 studies, grouped similar findings into 9 subthemes, and synthesized these into 4 overarching themes. The results of integration are shown in Figure 2.

#### 3.4.1 Overarching theme 1: negative emotional experiences toward illness and hemodialysis

Subtheme 1: Adjusting to illness and treatment posed challenges for young and middle-aged hemodialysis patients. In included qualitative studies, patients frequently indicated significant psychological distress and negative emotions linked to their physical challenges. Many patients also expressed despair about the future for themselves and their families, driven by concerns over the long-term impact of their illness, unpredictable disease progression, and variable prognosis. “After falling ill and undergoing continuous dialysis, my spouse felt the financial pressure at home was too great, and we divorced within 2 years. Alas, such was life (with tears in my eyes) (Male, 44, Primary school, Unemployed); While undergoing dialysis here, I occasionally contemplated the possibility of worse outcomes, fearing that the dialysis might not effectively remove toxins, which could then lead to infections affecting other organs (Female, 44, High school, Working)” (13). Patients indicated that fistulas and needle sites might lead to body image concerns and stigma. “Looking at their (established patients’) scars, I felt so scared (Female, 52)” (14). “Constant stares caused embarrassment and tears” (15). Some young patients denied kidney issues and dialysis need, resulting in treatment refusal; one patient stated: “I was reluctant to seek hospital treatment, as I could not believe there was a problem with my kidneys (Male, Unemployed) “(16). Patients indicated they felt aggressive over minor stressors and developed an aversion to dialysis machinery due to prolonged treatment; one patient said: “I detested the dialysis machinery, and thinking of dialysis caused depression (Male, Elementary, Unemployed) “(17). Additionally, patients experienced intense loneliness and boredom from living with the condition. A female patient expressed that: “I was deciding to kill myself, to be free from hemodialysis. I feel lonely. It is boring to live with such a condition. [She cried] (Cannot read and write, Merchant)” (18).

Subtheme 2: Young and middle-aged hemodialysis patients attempt to cope with negative emotions. Despite life dissatisfaction, some patients accepted their situation, comparing it favorably to graver illnesses. “Hemodialysis was better than facing death without it. Now, patients could stay with their families, avoiding direct succumbing to kidney disease (Female, 36)” (19). Eager for societal reintegration, a patient said: “Removing toxins through dialysis was good, but I hoped to find work; I was still young (Women, Middle school, Unemployed) “(13). “Post-dialysis, I aspired to a kidney transplant and a healthy, fulfilling life without illness or complications (Female, High school, Housewife)” (17). “Despite immense suffering from hemodialysis and kidney disease, the struggle fortified my resolve, allowing hopeful confrontation of reality (Male, Middle school, Unemployed)” (13).

#### 3.4.2 Overarching theme 2: experience of financial problems due to long-term hemodialysis treatment

Subtheme 3: Individual financial burden. As primary breadwinners, young and middle-aged patients undergoing long-term dialysis often face unemployment and hardship, struggling to cover basic needs. “Since my illness, I relied on my husband’s earnings. Limited by our rural income, our once school-attending children now worked to contribute financially” (20). “Thrice-weekly hemodialysis sessions, with their time constraints and health impacts, prevented me from securing steady employment or income (Female, 46, Unemployed)”; “Dialysis-induced weakness inhibited my ability to work, cutting off my income” (19). As a result, patients indicated that, desperate to afford the costly treatments, numerous patients were forced to sell their assets and seek financial assistance from family members and others (21).

Subtheme 4: Underdeveloped Areas’ Healthcare Policy Challenges. In a Pakistan study, patients indicated that financial constraints limited the access of low-income patients to hemodialysis, due to few available beds in government hospitals and high costs at private centers (22). Patients could not afford essential dialysis treatments due to facility scarcity (22). Similarly, In a Ghana study, patients indicated that the lack of government and insurance coverage for hemodialysis burdened them with the full treatment costs (16). “Migrant ‘hukou’ patients reported that despite policy relief for farmers, medical costs, including consultations, tests, and hospitalization, remain burdensome” (23).

#### 3.4.3 Overarching theme 3: breaking the normal life

Subtheme 5: Shifts in Family Dynamics. Disease and regular dialysis treatments were perceived by patients to decrease work capacity and strength, which in turn, was perceived to deteriorate their role as family pillars. Individuals undergoing hemodialysis indicated significant challenges in fulfilling their role responsibilities and managing family pressures (23). The rigid hemodialysis schedule and associated side effects were particularly disruptive to their parental roles, often impeding their ability to provide childcare and meet parental expectations. As a parent lamented: “Three four-hour weekly hospital visits broke my heart; I was absent for my child (Female, 41)”; Additionally, the authority in the family dynamic shifts, as one father explained, “My word used to be law, but now it is not.” (Male, 44) (8). Furthermore, patients described how illness prompted relinquishment of responsibilities, including transferring family finances to their wives and ceasing their sons’ recreational activities, while fatigue, a notable side effect, further impeded their parenting abilities (8). For instance, one parent recounted, “Post-dialysis exhaustion sometimes stopped me from listening to my child (Female, 43); fistula site pain weakened my arm, needing help to lift my son (Male, 28)” (8).

Patients indicated that discord in sexual life discord and challenges with pregnancy due to end-stage kidney disease impacted family life. They conveyed emotional struggles: “Dialysis during the day and my partner’s night work resulted in rare encounters and concern for our sparse sexual life” (24); “Lack of sexual desire for weeks might lead to affairs and family breakdowns” (25). Moreover, individuals undergoing hemodialysis indicated that menstrual and ovulation irregularities, reduced sexual function, and fertility challenges made pregnancy and motherhood seem unattainable for women on hemodialysis; the arteriovenous fistula’s physical mark and societal stigmatization further compounded these challenges, given the perception that only healthy women were suitable for motherhood (26). Additionally, they indicated that post-transplant pregnancies were short-lived due to kidney rejection and renal failure after childbirth, which necessitated the resumption of hemodialysis (8).

Subtheme 6: Changes in social roles. Qualitative findings revealed that altered body image was a significant factor contributing to social withdrawal among individuals undergoing hemodialysis. One female patient described her experience, saying: “Embarrassed and fearing questions, I avoided communication” (19). Moreover, patients indicated that inflexible dialysis schedule and limited energy forced them to narrow their social lives (23). For example, a patient stated: “A session consumed half my day, centering my life around dialysis” (20). In addition, individuals undergoing hemodialysis indicated that complications from dialysis significantly altered their social roles. As one male patient mentioned: “Fatigue reduced my ability to perform daily tasks, affecting social interactions and life quality (Secondary school, Merchant) “(18). Furthermore, symptoms like itching and restless legs syndrome discouraged my participation in social activities (27). Finally, “School attendance was challenging due to eight-hour weekly therapy sessions, causing frequent absences. Visible treatment effects, like an enlarged arm, and post-dialysis discomfort, sometimes also deterred me from attending” (28).

Subtheme 7: Behavior changes in daily life. Patients indicated that travel plans had to fit hemodialysis constraints, reducing destination options: “I used to avidly travel and enjoy sports, but my kidney condition put an end to those activities (Male, 63)” (19). Furthermore, dietary and drinking water restrictions imposed by dialysis often led to changes in social behaviors and daily routines, as described by patients: “Thirsty but unable to drink, disheartening (Male, 46)” (14). “Dietary restrictions from dialysis often lead me to avoid food at family gatherings (Male, 55)” (22).

#### 3.4.4 Overarching theme 4: patients are seeking multiple forms of support

Subtheme 8: Seeking professional knowledge support. Patients frequently sought explanations for their conditions and desired deeper knowledge of hemodialysis. For example, one patient said, “I used to use my spare time to consult the internet about nephropathy, internal fistulas, and dialysis” (24). However, patients often lacked awareness of kidney disease issues, especially those linked to hypertension or pregnancy. As a male patient mentioned, “I used to smoke, drink, and stay up late, ignoring warnings about kidney damage (College, Working)” (13). And patients indicated that they could identify recognized conditions such as hypertension or diabetes, but that kidney disease, which is often overlooked, was a complication (16). As a female patient shared, “My mother noticed my swelling, and despite our rural setting, she treated me with various medications. It wasn’t until I was 18 that I discovered my kidneys were impaired. Later, during pregnancy, I faced further issues, initially consulting a therapist. It was only on the day of my child’s birth that my condition deteriorated, prompting a visit to a doctor” (29).

Subtheme 9: Seeking family and social support. Patients indicated that they derived strength in life from their children and spouse. For example, one female patient said, “Her five children were her reason for living” (29) and another mentioned, “My family, including my husband, had been a pillar of support” (21). Additionally, with the help and support of friends and peers, patients gained relevant knowledge and improved their self-management skills. As a female patient noted, “Friends in the hemodialysis room provided significant support” (29), while a male patient shared, “Conversations with fellow patients offered valuable insights and treatment advice” (30). Furthermore, Community and support groups provided comfort and helped patients cope with challenges. A female patient expressed, “A community home turned me from fearful to optimistic” (30), and another patient said, “I encouraged other patients to join our support group, where they could receive valuable information and personalized assistance with their challenges” (8).

## 4 Discussion

The aim of this study is to explore the experiences, psychological states and needs of young and middle-aged hemodialysis patients (aged 18–65 years) through a meta-synthesis of qualitative studies. Our findings suggest that patients face negative emotional experiences toward illness and hemodialysis, experience of financial problems due to long-term hemodialysis treatment, disruptions to normal life, and struggle to seek multiple forms of support. Compared with the existing literature, this study is the first to systematically summarize the experiences and needs of young and middle-aged hemodialysis patients, offering insights for better patient-centered care and disease management in clinical practice. Based on these findings, we propose the following recommendations.

### 4.1 Pay attention to the inner world of young and middle-aged hemodialysis patients and guide positive psychological experience

Young and middle-aged individuals, who shoulder the highest work responsibilities and family/societal obligations, endure substantial physical and psychological trauma from kidney disease and hemodialysis. This leads to physiological decline, increased complications, symptom burden, and social dysfunction. Patients often experience a variety of negative emotions, including self-blame, regret, fear, anxiety, pain, irritability, disgust, loneliness, depression, despair, and suicidal thoughts. Some may even refuse treatment. However, they also attempt to cope with negative emotions. For customized nursing interventions, medical staff should maintain consistent communication, promptly assess the psychological states of patients and identify early signs of negative emotions. For patients who already have severe emotional distress, it is essential to facilitate the expression of feelings, offer effective psychological counseling, support self-adjustment, and encourage a positive outlook. Psychological interventions, such as humor therapy (31), mindfulness-based stress reduction (32), and cognitive-behavioral therapy (33) help individuals to change maladaptive cognitions and behaviors, alleviate negative emotions, and develop coping mechanisms. More deeply, the results of this study showed that most patients had negative emotions due to symptom burden, decreased physical function, and disease management strategies, such as multidisciplinary nursing care, which promotes collaboration among healthcare professionals, ensuring optimal treatment, maximal symptom relief, and the reduction of negative emotions (34).

### 4.2 Ease the economic burden

The study showed that hemodialysis patients face significant financial strain from treatment costs, symptom management, unemployment, and healthcare policies. To alleviate their burden, the following actions are recommended. While systemic reforms require governmental action, medical staff could implement immediate interventions to mitigate economic burdens. Clinicians could proactively identify patients’ financial distress and establish confidential counseling channels for those opting to disclose economic concerns. This could help in identifying potential financial resources and family support systems available to patients for long-term hemodialysis. Tailoring dialysis regimens to individual clinical profiles and residual renal function may yield both medical and socioeconomic benefits. Incremental hemodialysis, offering sessions of 3–4 h ≤2 times per week or <3 h ≥3 times, adapts to personal situations and allows flexible treatments adjustments, which can slow the renal function decline, reduce dialysis frequency, lower medical costs and ease patients’ financial strain (35, 36). Introducing nocturnal dialysis services and implementing flexible scheduling options, such as home hemodialysis, support patients’ employment needs and increase their income. Through the implementation of these clinically grounded, patient-oriented strategies, to alleviate the economic burdens inherent in mostly long-term hemodialysis management.

### 4.3 Assist the patient to gradually return to normal life

The prolonged hemodialysis process, with its specialized treatment and symptom burden, disrupts patients’ lives. Hemodialysis patients often experience reduced family function due to dialysis complications and disease treatment needs. Healthcare providers should take a family-centered approach, wherein interventions engage both patients and their family members as collaborative partners in treatment. This approach involves structured family education programs detailing renal pathophysiology, dialysis frequency protocols, evidence-based dietary modifications, and pharmacological management, etc. (37). By strengthening familial health literacy and caregiving capacity, such interventions may reduce treatment-related complications while enhancing psychosocial support systems to facilitate successful household reintegration (37). Medical staff could communicate more with patients and guide them through interventions such as hand-foot massage (38), aerobic exercise (39), and self-acupressure (40) to alleviate symptom cluster, enhance their quality of life, and facilitate return to normal life. For patients pursuing vocational or educational reintegration, care teams should develop tailored transition plans featuring flexible dialysis scheduling coordinated with occupational rehabilitation specialists. This multidimensional care framework aims to optimize both clinical outcomes and psychosocial adaptation throughout the disease trajectory. Hemodialysis patients require dietary restriction due to their severely impaired kidney function, which prevents them from properly excreting metabolic waste and regulating electrolyte and water balance (41, 42). However, the results of this study indicate that dietary restrictions in young and middle-aged hemodialysis patients often lead to decreased appetite, loss of social function, and reduced quality of life. Healthcare teams should collaborate with patients to co-develop individualized dietary plans, integrating clinical parameters and psychosocial needs. This evidence-based approach aims to reduce unnecessary dietary constraints while supporting social reintegration through nutrition autonomy.

### 4.4 Providing hemodialysis patients with essential knowledge, peer and community support

The results of this study show that many patients are uninformed about their disease’s origins and self-management, highlighting the need for better health education. To empower patients, medical staff could develop a series of educational materials, including brochures, posters, videos, and science communication via digital platforms. These resources would disseminate knowledge on kidney disease self-management and dialysis, ultimately improving patients’ health literacy and self-care capabilities (43, 44). The results of this study suggest that hemodialysis patients often need to seek peer and social support to help them better face the disease and the challenges of dialysis treatment. Therefore, medical staff could help patients to establish peer support network to achieve the purpose of returning to society and promoting patients’ trust (45). The community could organize recreational activities for dialysis patients to participate in (such as painting, calligraphy classes, and light exercise classes) to enhance their sense of social integration.

## 5 Conclusion

This study conducted a meta-synthesis of 22 qualitative studies to comprehensively analyze and interpret the actual experiences, psychological states, and needs of young and middle-aged hemodialysis patients. The findings showed that young and middle-aged hemodialysis patients face negative emotional experiences toward illness and hemodialysis, experience of financial problems due to long-term hemodialysis treatment, disruptions to normal life and seeking multiple forms of support. Noting the rising number of young hemodialysis patients, the study emphasizes their psychological journey and specific needs. Healthcare providers should attach importance to this group, to meet their specific needs, and support their active recovery. The goal is to help them relieve negative emotions, reduce financial burden, return to normal life and meet support-seeking behaviors.

## 6 Limitations

(1) Most literature quality evaluations rated grade B, with one grade A study, indicating a moderate overall quality. (2) The literature search included only Chinese and English sources, excluding those in other languages. (3) The study covered 14 countries with varying economic levels and nursing environments, reflecting a broad heterogeneity, the findings of this review may not be universally applicable to medical staff across all countries.

## Data Availability

The original contributions presented in the study are included in the article/Supplementary material, further inquiries can be directed to the corresponding author.
