# Supplementary material for: Real experience of young and middle-aged hemodialysis patients: a qualitative meta-synthesis
Source: Front Med (Lausanne). 2025 Apr 7;12:1530465. doi: 10.3389/fmed.2025.1530465 (PMC12009844; doi:10.3389/fmed.2025.1530465)
Supplement: Supplementary file 1 [file Data_Sheet_1.pdf]

Tong et al. BMC Medical Research Methodology 2012, 12:181
http://www.biomedcentral.com/1471-2288/12/181

BMC Medical Research Methodology

CORRESPONDENCE Open Access

Enhancing transparency in reporting the synthesis of qualitative research: ENTREQ

Allison Tong[1,2*†], Kate Flemming[3†], Elizabeth McInnes[4†], Sandy Oliver[5] and Jonathan Craig[1,2]

Abstract

Background: The syntheses of multiple qualitative studies can pull together data across different contexts, generate new theoretical or conceptual models, identify research gaps, and provide evidence for the development, implementation and evaluation of health interventions. This study aims to develop a framework for reporting the synthesis of qualitative health research.

Methods: We conducted a comprehensive search for guidance and reviews relevant to the synthesis of qualitative research, methodology papers, and published syntheses of qualitative health research in MEDLINE, Embase, CINAHL and relevant organisational websites to May 2011. Initial items were generated inductively from guides to synthesizing qualitative health research. The preliminary checklist was piloted against forty published syntheses of qualitative research, purposively selected to capture a range of year of publication, methods and methodologies, and health topics. We removed items that were duplicated, impractical to assess, and rephrased items for clarity.

Results: The Enhancing transparency in reporting the synthesis of qualitative research (ENTREQ) statement consists of 21 items grouped into five main domains: introduction, methods and methodology, literature search and selection, appraisal, and synthesis of findings.

Conclusions: The ENTREQ statement can help researchers to report the stages most commonly associated with the synthesis of qualitative health research: searching and selecting qualitative research, quality appraisal, and methods for synthesising qualitative findings. The synthesis of qualitative research is an expanding and evolving methodological area and we would value feedback from all stakeholders for the continued development and extension of the ENTREQ statement.

Keywords: Thematic synthesis, Standards, Qualitative health research, Reporting

Background

Methods to synthesise qualitative research began with the recognition that providing evidence-based healthcare and health policy requires a range of evidence beyond that provided by the 'rationalist' model of systematic reviewing of quantitative research [1]. Qualitative research aims to provide an in-depth understanding into human behaviour, emotion, attitudes and experiences. The synthesis of findings from multiple qualitative studies can provide a range and depth of meanings, experiences, and perspectives of participants across health-care contexts. Syntheses of qualitative research can pull together data across different contexts, generate new theoretical or conceptual models, identify research gaps, inform the development of primary studies, and provide evidence for the development, implementation and evaluation of health interventions [2-9]. The synthesis, or "bringing together" of the findings of primary qualitative studies is emerging as an important source of evidence for healthcare and policy [10]. Many aspects of the methods for synthesising qualitative research are in the early stages of development.

The number of published syntheses of qualitative health research is increasing (Figure 1). There are a wide range of qualitative synthesis methods with many common features, but also key differences [1]. The

* Correspondence: allison.tong@sydney.edu.au
†Equal contributors
[1]Sydney School of Public Health, University of Sydney, Sydney, NSW 2006, Australia
[2]Centre for Kidney Research, The Children's Hospital at Westmead, Westmead, NSW 2145, Australia
Full list of author information is available at the end of the article

BioMed Central

© 2012 Tong et al.; licensee BioMed Central Ltd. This is an Open Access article distributed under the terms of the Creative Commons Attribution License (http://creativecommons.org/licenses/by/2.0), which permits unrestricted use, distribution, and reproduction in any medium, provided the original work is properly cited.

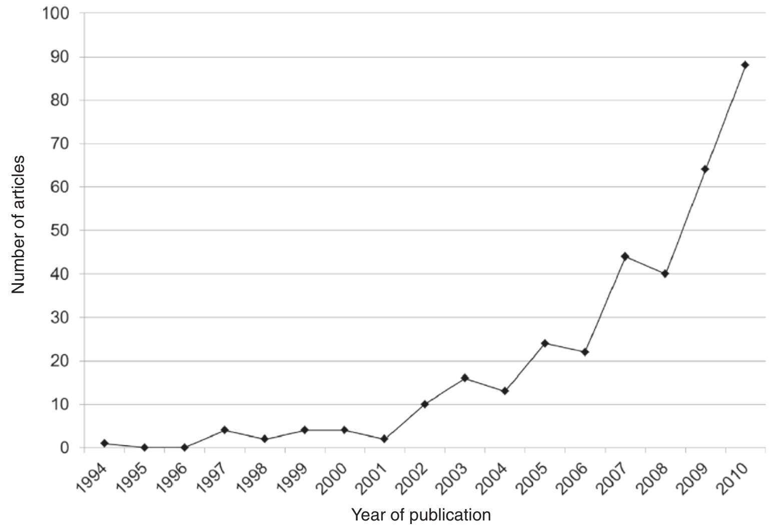

Figure 1 Number of published synthesis of qualitative health research.

main methods of qualitative synthesis include: meta-ethnography [11]; thematic synthesis [12]; critical interpretive synthesis [4]; narrative synthesis [13]; and meta-study [14-16]. One of the first methods identified for synthesising qualitative research - meta-ethnography - has subsequently influenced the development of other methods such as thematic analysis and critical interpretive synthesis through the use of its terminology and concepts, as well as extending and adapting its methods. Figure 2 provides examples of the wide-ranging terms used to describe different qualitative synthesis methods. Some of the adaptations of qualitative syntheses have, however, resulted in inconsistent use of terms for describing key stages of synthesis [17]. For users of qualitative syntheses the different labels used to describe similar qualitative synthesis methods and the inconsistent use of terms to describe the different stages within qualitative reviews can be confusing [1,18]. While there are differences in approaches and rationale for some qualitative synthesis methods (for example, Critical Interpretive Synthesis may be better suited for large diverse bodies of literature while meta-ethnography may be better for analysing a smaller number of papers) [4] there is a core set of techniques common to most qualitative synthesis methods.

While there are reporting guidelines for qualitative research [19], there are no published guidelines for *reporting* the synthesis of qualitative research. Reporting guidelines

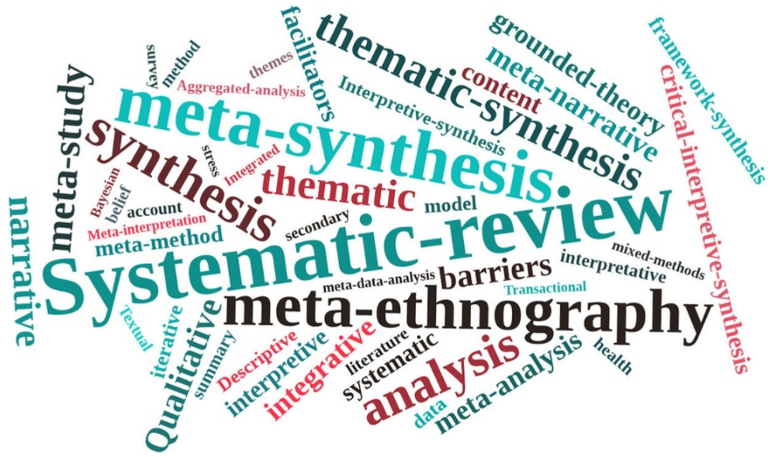

Figure 2 Word cloud of the methodological terms used in published synthesis of qualitative health research. Word clouds give more prominence to words that appear more frequently in the source text. The methodological terms were extracted from the title/abstract/full text of 381 published synthesis of qualitative health research (to 31st May 2011) and entered into Tagxedo, an online tool which generated a word cloud.

for quantitative systematic reviews exist and these are helping to set standards for both the conduct and reporting of these reviews [20]. Currently, most syntheses of qualitative research have been undertaken by those with an interest in methodological development, and therefore reviews appear to be well-reported. Increasingly, the methodologies associated with synthesis of qualitative research are being used by researchers and students new to the process. It is important at this time to begin to establish reporting standards. Developing reporting guidelines for qualitative synthesis may assist researchers to improve both the conduct and reporting of qualitative syntheses and enable the end-user to better understand the processes involved in developing a qualitative synthesis.

The aim of this paper is to report on the first phase of the development of guidelines to encourage transparency in reporting syntheses of qualitative research; to assist end-users to identify the core steps involved and to provide a tool to help clarify to the various concepts and terms used to describe similar processes in qualitative syntheses.

Methods

ENTREQ Development

It is acknowledged that there is no single best or correct approach to developing guidelines [21]. Where feasible, we have reported the development of our guideline drawing from steps provided in 'Guidance for developers of health research reporting guidelines' by David Moher and colleagues [21], available at www.equator-network.org (an international initiative that seeks to improve reliability and value of medical research by promoting transparent reporting).

Identify the need for a guideline

We identified the need for a reporting guideline for syntheses of qualitative research as a result of our collective experiences in using, publishing, reviewing and teaching syntheses of qualitative health research, debriefing notes taken after an international conference symposium on the synthesis of qualitative health research (Qualitative Health Research Conference in Vancouver, Canada, 2010, KF/AT) and a seminar at the Qualitative Health Research Collaboration in Sydney, Australia, 2011, (AT/EM).

To further establish a need for a reporting guideline, we conducted a comprehensive search for guidance and reviews relevant to the synthesis of qualitative research, methodology papers, and published syntheses of qualitative health research using the terms for "qualitative research" combined with terms relating to synthesis (systematic review, synthesis, thematic synthesis, meta-ethnography, meta-study, meta-analysis) (Additional file 1). The searches were conducted in electronic medical literature databases including MEDLINE, EMBASE, and CINAHL from inception to 20th May 2011, and in Google Scholar. Relevant organisational websites including the EQUATOR Network database of reporting guidelines (www.equator-network.org) and reference lists of relevant articles were also searched. We identified 381 syntheses of qualitative research, with the number of publications exponentially increasing from 1994 to May 2011 (Additional file 2, Figure 1).

Generating items for inclusion in the checklist

The initial items for inclusion in the preliminary "Enhancing transparency in reporting the synthesis of qualitative research (ENTREQ) Statement" were generated inductively from guides to synthesising qualitative health research [1,10], seminal methodology papers [4,11,12,22-24] and the authors' experience in conducting and appraising qualitative syntheses (AT, KF, EM,). The items were compiled and grouped into five categories: introduction; methods and methodology; literature search and selection; appraisal; and synthesis of findings.

Pilot testing the checklist

In order to test our preliminary framework and to reach consensus for the inclusion of each item, the reporting framework was pilot tested against forty published syntheses of qualitative research, which were purposively selected from our search results to capture a range of year of publication, methods and methodologies, and health topics (Additional file 3). Three members of the research team (AT/KF/EM) independently piloted the guidance initially against 32 of these reviews, by extracting relevant data for each guidance item. During this time we met via teleconferences to discuss the results of the testing and made a series of revisions to the ENTREQ Statement. We removed items that were duplicated. Items were also rephrased for clarity where there was ambiguity. The revised statement was tested against the eight remaining reviews and no further changes were made. On average, it took 5 to 20 minutes to assess each review using the ENTREQ Statement. The results are provided in Additional file 3.

Results and discussion

ENTREQ Statement: content and rationale

The ENTREQ statement consists of 21 items grouped into five main domains: introduction, methods and methodology, literature search and selection, appraisal, and synthesis of findings (Table 1). For each item, a descriptor and examples are provided. Below we present a rationale for each domain and its associated items.

Introduction, methods and methodology (Domains 1 and 2)

The methodology and approaches selected are usually influenced by the research question (outlined in the

Table 1 Enhancing transparency in reporting the synthesis of qualitative research: the ENTREQ statement

No	Item	Guide and description
**1**	Aim	State the research question the synthesis addresses.
**2**	Synthesis methodology	Identify the synthesis methodology or theoretical framework which underpins the synthesis, and describe the rationale for choice of methodology *(e.g. meta-ethnography, thematic synthesis, critical interpretive synthesis, grounded theory synthesis, realist synthesis, meta-aggregation, meta-study, framework synthesis).*
**3**	Approach to searching	Indicate whether the search was pre-planned (*comprehensive search strategies to seek all available studies)* or iterative (*to seek all available concepts until they theoretical saturation is achieved).*
**4**	Inclusion criteria	Specify the inclusion/exclusion criteria *(e.g. in terms of population, language, year limits, type of publication, study type).*
**5**	Data sources	Describe the information sources used (e.g. *electronic databases (MEDLINE, EMBASE, CINAHL, psycINFO, Econlit), grey literature databases (digital thesis, policy reports), relevant organisational websites, experts, information specialists, generic web searches (Google Scholar) hand searching, reference lists)* and when the searches conducted; provide the rationale for using the data sources.
**6**	Electronic Search strategy	Describe the literature search *(e.g. provide electronic search strategies with population terms, clinical or health topic terms, experiential or social phenomena related terms, filters for qualitative research, and search limits).*
**7**	Study screening methods	Describe the process of study screening and sifting *(e.g. title, abstract and full text review, number of independent reviewers who screened studies).*
**8**	Study characteristics	Present the characteristics of the included studies *(e.g. year of publication, country, population, number of participants, data collection, methodology, analysis, research questions).*
**9**	Study selection results	Identify the number of studies screened and provide reasons for study exclusion *(e,g, for comprehensive searching, provide numbers of studies screened and reasons for exclusion indicated in a figure/flowchart; for iterative searching describe reasons for study exclusion and inclusion based on modifications t the research question and/or contribution to theory development).*
**10**	Rationale for appraisal	Describe the rationale and approach used to appraise the included studies or selected findings *(e.g. assessment of conduct (validity and robustness), assessment of reporting (transparency), assessment of content and utility of the findings).*
**11**	Appraisal items	State the tools, frameworks and criteria used to appraise the studies or selected findings *(e.g. Existing tools: CASP, QARI, COREQ, Mays and Pope* [25]; *reviewer developed tools; describe the domains assessed: research team, study design, data analysis and interpretations, reporting).*
**12**	Appraisal process	Indicate whether the appraisal was conducted independently by more than one reviewer and if consensus was required.
**13**	Appraisal results	Present results of the quality assessment and indicate which articles, if any, were weighted/excluded based on the assessment and give the rationale.
**14**	Data extraction	Indicate which sections of the primary studies were analysed and how were the data extracted from the primary studies? *(e.g. all text under the headings "results /conclusions" were extracted electronically and entered into a computer software).*
**15**	Software	State the computer software used, if any.
**16**	Number of reviewers	Identify who was involved in coding and analysis.
**17**	Coding	Describe the process for coding of data *(e.g. line by line coding to search for concepts).*
**18**	Study comparison	Describe how were comparisons made within and across studies *(e.g. subsequent studies were coded into pre-existing concepts, and new concepts were created when deemed necessary).*
**19**	Derivation of themes	Explain whether the process of deriving the themes or constructs was inductive or deductive.
**20**	Quotations	Provide quotations from the primary studies to illustrate themes/constructs, and identify whether the quotations were participant quotations of the author's interpretation.
**21**	Synthesis output	Present rich, compelling and useful results that go beyond a summary of the primary studies (e.g. *new interpretation, models of evidence, conceptual models, analytical framework, development of a new theory or construct).*

introduction), intended synthesis output, reviewer's philosophical position, context, and target audience. Also, reviewers may choose their approach according to the type of data available. For example, meta-ethnography works well with primary qualitative studies offering "thick descriptions" and in-depth analysis. Thematic synthesis is possible with "thinner" studies. A recent review of qualitative syntheses found that nine main approaches were used to synthesise qualitative research including: critical interpretive synthesis, grounded theory synthesis, meta-ethnography, meta-study, thematic synthesis, meta-narrative synthesis, textual narrative synthesis, framework synthesis, and ecological triangulation [1]. A summary of commonly used approaches for synthesising qualitative health research is provided in Table 2.

Literature search and selection (Domain 3)

Conducting a systematic search which is reproducible and comprehensive is a distinguishing characteristic of a

Table 2 Summary of common methodologies for the synthesis of qualitative health research*

Methodology	Critical interpretive synthesis	Grounded theory synthesis	Meta-ethnography	Meta-study	Thematic synthesis
**Key seminal methodology references**	Dixon-woods et al. 2006 [4]	Kearney 2001 [23], Eaves 2001 [22]	Noblit and Hare 1988 [11], Britten et al. 2002 [2]	Paterson et al. 2001 [24]	Thomas and Harden 2008 [12]
**Philosophical positioning****	*Subjective idealism* – no single shared reality independent of multiple alterative human constructions	*Objective idealism* – a world of collectively shared understandings exists	*Objective idealism* – a world of collectively shared understandings exists	*Subjective idealism* – no single shared reality independent of multiple alterative human constructions	*Critical realism* – knowledge of reality is medicated by one's beliefs and perspectives
**Literature search**	Theoretical sampling	Theoretical sampling	Non-specified	Not-specified	Systematic, comprehensive
**Quality appraisal**	The degree to which the research findings can inform theory development	Implicit judgement about the context, quality and usefulness of the study	Judgement based on relevance; CASP	Focuses on rigour and the epistemological soundness of the research methods	Criteria related to aims, context, rationale, methods and findings, reliability, validity, appropriateness of methods for ensuring findings are grounded in participant perspectives
**Analysis techniques and concepts**	• Concurrent iteration of the research questions	• Concurrent data collection and analysis	• Reciprocal translational analysis (translation of concepts from individual studies – 1st/2nd order constructs)	• Analyse findings – meta-data-analysis	• Line by line coding of text from primary studies
	• Extract data and summarise papers	• Theory is derived inductively from the data	• Refutational synthesis (explore and explain contradictions between studies – 1st/2nd order constructs)	• Analyse methods – meta-method)	• Free codes organised into descriptive themes
	• Define and apply codes	• Constant comparison of data	• Lines of argument (grounded theorising based on synthesising translations)	• Analyse theory – meta-theory	• Further interpretation to develop analytical themes
	• Develop a critique, generate themes			• Bring together all three components of the analysis	
**Synthesis output**	• New theoretical conceptualisation – synthetic construct	• Generation of a new, higher-level grounded theory	• New insights – 3rd order constructs	• Account for differences in research findings	• Analytical themes that offer a new interpretation that goes beyond the primary studies
				• New interpretation of phenomena studied	
**Topic areas and study references†**	Access to healthcare by vulnerable groups [4], pain management [26]	Domestic violence [23], caregiving [22]	Medicine-taking [3], patients' help-seeking experiences in cancer presentation [6], palliative care [27]	Chronic illness experience [14], influences on shared decisions making [15], adolescent health [16]	Children's experiences of health eating [12], chronic kidney disease [28], people's understanding of cancer risk [29], organ transplantation [7], patient-physician relationships [30]

*This is not a complete list of methodologies as methodologies for the synthesis of qualitative health research are wide ranging; **Adapted from Barnett-Page and Thomas [1] and Spencer et al. [31]. †References selected to reflect a range of topic areas in health research.

systematic review; however there are few developed and tested methods for locating qualitative research, and lack of consensus as to whether systematic searching is required [32]. Some argue that exhaustive searching is not necessary. Instead, reviewers may adopt an iterative approach where all the available concepts rather than studies are sought until saturation is reached [1].

A pre-planned sensitive search strategy may combine search terms relating to the population and context, with those relating to the health or clinical topic, and terms relating to experiential and social phenomena (such as knowledge, attitudes, beliefs, understanding, preferences, perspectives). These can then be combined with terms for qualitative methods and methodology. Methodological filters for qualitative research have been developed but have undergone little replication and validation [32]. There are also differences in the indexing of qualitative research within electronic databases such as

MEDLINE, EMBASE, PsycINFO and CINAHL. Within published syntheses of qualitative research there is often a lack of transparency about the search processes employed, with neither the search strategy nor databases detailed [33]. For a comprehensive approach, the PRISMA flowchart is recommended for reporting the different phases of searching, screening and identifying studies for inclusion in the qualitative synthesis [20]. Qualitative research can often be found in the grey literature (e.g. technical reports, working papers, thesis publications). To locate relevant studies, reviewers can search relevant organisational websites, Google Scholar, thesis databases, specialist journals, and consult with experts (researchers, providers, policy makers) in the relevant fields and librarians.

The inclusion and exclusion of studies may be defined by factors including population characteristics, health or clinical topic, methods and methodology (philosophical approach), language, time frame, or type of publication; and this should be justified. For readers to make an assessment about the transferability of the findings to their own setting, a description of the study characteristics, screening process, and reasons for excluding studies is needed.

Appraisal (Domain 4)

Quality assessment of qualitative research is challenging and contentious [25]. Just as there are no standardised criteria for assessing the quality of all quantitative research, standardising criteria for assessing the standard of conduct in all qualitative research which embraces a range of designs, is not possible or appropriate [31,34]. Also, there is little evidence on how the quality of reporting reflects the robustness, trustworthiness and transferability of the findings of qualitative studies [35]. Nevertheless, most published syntheses of qualitative research include a quality appraisal of the primary studies. The rationale underpinning quality assessment and the methods used to appraise quality vary widely but can be broadly characterised into three approaches: assessment of study conduct, appraisal of study reporting, and implicit judgement of the content and utility of the findings for theory development. Some syntheses exclude low quality studies, while others comment on or weight study findings according to their quality [36].

Several appraisal tools have been used including the Critical Appraisal Skills Program (CASP) [37] which addresses the principles and assumptions underpinning qualitative research but does not claim to be a definitive guide; the Qualitative Assessment Review Instrument Tool (QARI) [38], which suggests general questions that require the reader to make a judgement for example about the "congruency" of the research methodology with the state philosophical perspective, research questions, data collection, interpretation of the results; and Consolidated Criteria for Reporting Qualitative Research (COREQ) [19] which is the only framework developed explicitly for assessing reporting. Some reviewers have developed their own appraisal framework selecting items from existing criteria [25,39-41], augmented with additional criteria they deemed were specifically relevant to the research topic. These were usually identified by discussion and consensus among the research group. For example, Brunton et al. [42] conducted a systematic review of qualitative research on children and physical activity and used existing criteria proposed for assessing quality of qualitative research but included an additional item, "actively involved children to an appropriate degree in the design and conduct of the study," which they deemed relevant to their review [42].

Systematic reviewers of qualitative studies have found that many primary qualitative studies are poorly reported [3]. Also, some reviewers have found that studies with sparse detail about the conduct of the research tend to contribute less to the synthesis [28]. An assessment of the quality of reporting can allow readers to make an informed judgement about the credibility (can the research findings be trusted?), dependability (is the process of research logical, traceable and clearly documented?), transferability (are the research findings relevant to other settings?) and confirmability (are the research findings and interpretations linked to the data?). A reporting framework can also function as a screening tool for systematic reviewers to determine study eligibility and inform the development of future qualitative studies on the topic of interest. For example, it can highlight qualitative methods and methodologies that have been effective in gaining in-depth insight into participants' perspectives, beliefs and attitudes, and identify those which could generate more understanding about a phenomena, but have not been tried and tested. Also, the process of appraisal can facilitate a deeper understanding of included papers.

Existing frameworks for reporting qualitative research may be considered and used as a starting point and adapted to suit the synthesis topic. The framework should capture the range of methods and methodologies of the included studies. In some instances, multiple reviewers have independently assessed quality and discussed quality appraisal to achieve consensus. Also, the rationale for weighting or excluding studies based on quality appraisal should be explicit.

Synthesis of findings (Domain 5)

For clarity of reporting the analysis process, reviewers should define which sections of the included articles were actually analysed; and describe the process of

coding, comparing and interpreting the data. Specific analysis techniques and concepts are provided in Table 2. Details about use of software and number of reviewers involved in coding and analysis can allow readers to assess the dependability of the findings. It enables readers to assess whether data are managed in a systemic way. Quotations from the articles may be included to illustrate the themes or constructs identified. The target audience should also be considered when reporting and presenting the synthesis output. Ultimately, the synthesis should generate rich, compelling and new insights that go beyond a summary of the primary studies; however some "implicit judgment" and team discussion may be required to assess this.

Conclusions

The ENTREQ statement was developed to promote explicit and comprehensive reporting of the synthesis of qualitative studies. We acknowledge it is unlikely that a standardised set of procedures will ever be developed, more probably, a 'methodological palette' will be created from which reviewers can draw methods relevant to the focus of their review [9]. The proposed guidelines covers reporting items relating to methodology and methods, literature searching and selection, appraisal and the synthesis of findings.

The purpose of the ENTREQ statement is to offer guidance for researchers and reviewers to improve the reporting of synthesis of qualitative health research. We believe this document can be a useful resource and reference for those learning how to conduct a synthesis of qualitative research and readers of syntheses of qualitative health research. But we emphasise that this is not an absolute, definitive framework. Also, we acknowledge that we did not complete a Delphi exercise as recommended by the "Guidance for developers of health research reporting guidelines" [21] due to resource limitations. However, we believe that this initial development of the ENTREQ Statement is a crucial step for the development of a Delphi exercise.

We encourage authors to evaluate the checklist to assess whether it is useful for improving the completeness of reporting the synthesis of qualitative research. The synthesis of qualitative research is an expanding and evolving methodological area and we would value feedback from all stakeholders for the continued development and extension of the ENTREQ statement in terms of content, clarity and feasibility.

Additional files

Additional file 1: Search strategy.

Additional file 2: Search results.

Additional file 3: Pilot test: assessment of 40 published synthesis of qualitative research using the ENTREQ Statement.

Abbreviations

ENTREQ: Enhancing transparency in the reporting of qualitative health research.

Competing interests

The authors declare that they have no competing interests.

Authors' contributions

AT, KF, EM collected and analysed the data. AT/KF/EM drafted the manuscript. All authors critically reviewed and provided intellectual input on the manuscript. All authors read and approved the final manuscript.

Acknowledgments

AT is supported by the National Health and Medical Research Council (NHMRC) Fellowship [ID1037162].

Author details

[1]Sydney School of Public Health, University of Sydney, Sydney, NSW 2006, Australia. [2]Centre for Kidney Research, The Children's Hospital at Westmead, Westmead, NSW 2145, Australia. [3]Department of Health Sciences, University of York, Heslington, York Y010 5DD, UK. [4]National Centre for Clinical Outcomes Research, Australian Catholic University, St Vincent's Hospital, Darlinghurst, NSW 2010, Australia. [5]Institute of Education, University of London, London WC1H 0AL, UK.

Received: 25 June 2012 Accepted: 12 November 2012
Published: 27 November 2012

References

1. Barnett-Page E, Thomas J: *Methods for the synthesis of qualitative research: a critical review*. 01/09th edition. London: ESRC National Centre for Research Methods, Social Science Research Unit, Institute of Education, University of London; 2009.
2. Britten N, Campbell R, Pope C, Donovan J, Morgan M, Pill R: **Using meta ethnography to synthesise qualitative research: a worked example.** *J Health Serv Res Policy* 2002, **7**(4):209–215.
3. Campbell R, Pound P, Pope C, Britten N, Pill R, Morgan M, Donovan J: **Evaluating meta-ethnography: a synthesis of qualitative research on lay experience of diabetes and diabetes care.** *Soc Sci Med* 2003, **56**(4):671–684.
4. Dixon-Woods M, Cavers D, Agarwal S, Annandale E, Arthur A, Harvey J, Hsu R, Katbamna S, Olsen R, Smith L, *et al*: **Conducting a critical interpretive synthesis of the literature on access to healthcare by vulnerable groups.** *BMC Med Res Methodol* 2006, **6**:35–47.
5. Pound P, Britten N, Morgan M, Yardley L, Pope C, Daker-White G, Campbell R: **Resisting medicines: a synthesis of qualitative studies of medicine taking.** *Soc Sci Med* 2005, **61**(1):133–155.
6. Smith LK, Pope C, Botha JL: **Patients' help-seeking experiences and delay in cancer presentation: a qualitative synthesis.** *Lancet* 2005, **366**:825–831.
7. Tong A, Morton RL, Howard K, Craig JC: **Adolescent experiences following organ transplantation: a systematic review of qualitative studies.** *J Pediatr* 2010, **155**(4):542–549.
8. McInnes RJ, Chambers JA: **Supporting breastfeeding mothers: qualitative synthesis.** *J Adv Nurs* 2008, **62**(4):407–427.
9. Flemming K: **The use of morphine to treat cancer-related pain: a synthesis of quantitative and qualitative research.** *J Pain Symptom Manag* 2010, **39**(1):139–154.
10. Ring N, Ritchie K, Mandava L, Jepson R: *A guide to synthesising qualitative research for researchers undertaking health technology assessments and systematic reviews*. 2010. Available from: http://www.nhshealthquality.org/nhsqis/8837.html.
11. Noblit GW, Hare HD: *Meta-ethnography: synthesising qualitative studies*. Thousand Oaks: Sage; 1988.
12. Thomas J, Harden A: **Methods for the thematic synthesis of qualitative research in systematic reviews.** *BMC Med Res Methodol* 2008, **8**:45.
13. Arai L, Britten N, Popay J, Roberts H, Petticrew M, Rodgers M, Sowden A: **Testing methodological developments in the conduct of narrative**

synthesis: a demonstration review of research on the implementation of smoke alarm interventions. *Evid Policy* 2007, **3**(3):361–383.
14. Thorne S, Paterson B, Acorn S, Canam C, Joachim G, Jillings C: **Chronic illness experience: insights from a meta-study.** *Qual Health Res* 2002, **12:**437–452.
15. Edwards M, Davies M, Edwards A: **What are the external influences on information exchange and shared decision-making in healthcare consultations: a meta-synthesis of the literature.** *Patient Educ Counsel* 2009, **75:**37–52.
16. Timminen KA, Holt NL: **A meta-study of qualitative research examining stressor appraisals and coping among adolescents in sport.** *J Sports Sci* 2010, **28**(14):1563–1580.
17. Atkins S, Lewin S, Smith H, Engel M, Fretheim A, Volminck J: **Conducting a meta-ethnography of qualitative literature: lessons learnt.** *BMC Med Res Methodol* 2008, **8:**21.
18. Dixon-Woods M: **Using framework-based synthesis for conducting reviews of qualitative studies.** *BMC Med* 2011, **9:**39.
19. Tong A, Sainsbury P, Craig JC: **Consolidated criteria for reporting qualitative research (COREQ): a 32-item checklist for interviews and focus groups.** *Int J Qual Health Care* 2007, **19**(6):349–357.
20. Moher D, Liberati A, Tetzlaff J, Altman D, The PRISMA Group: **Preferred reporting items for systematic reviews and meta-analyses: The PRISMA Statement.** *PLOS Med* 2009, **6**(7):e1000097.
21. Moher D, Schulz KF, Simera I, Altman D: **Guidance for developers of health research reporting guidelines.** *PLOS Med* 2010, **7**(2):e1000217. doi:1000210.1001371/journal.pmed.1000217.
22. Eaves YD: **A synthesis technique for grounded theory data analysis.** *J Adv Nurs* 2001, **35**(5):654–663.
23. Kearney MH: **Enduring love: a grounded formal theory of women's experience of domestic violence.** *Res Nurs Health* 2001, **24:**270–282.
24. Paterson BL, Thorne SE, Canam C, Jillings C: *Meta-study of qualitative health research. A practical guide to meta-analysis and meta-synthesis.* Thousand Oaks: Sage; 2001.
25. Mays N, Pope C: **Qualitative research in health care: assessing quality in qualitative research.** *Br Med J* 2000, **320:**50–52.
26. Flemming K: **The use of morphine to treat cancer-related pain: a synthesis of quantitative and qualitative research.** *J Pain Symptom Manage* 2010, **39**(1):139–154.
27. Hubbard G, McLachlan K, Forbat L, Munday D: **Recognition by family members that relatives with neurodegenerative disease are likely to die within a year: A meta-ethnography.** *Palliat Med* 2011, Online first.
28. Morton RL, Tong A, Howard K, Snelling P, Webster AC: **The views of patients and carers in treatment decision-making for chronic kidney disease: systematic review and thematic synthesis of qualitative studies.** *Br Med J* 2010, **340:**c112.
29. Lipworth WL, Davey H, Carter S, Hooker C: **Beliefs and beyond: what can we learn from qualitative studies of lay people's understanding of cancer risk.** *Health Expect* 2010, **13**(2):113–124.
30. Ridd M, Shaw A, Lewis G, Salisbury C: **The patient-doctor relationship: a synthesis of the qualitative literature on patients' perspectives.** *Br J Gen Prac* 2009, **29**(561):e116–e133.
31. Spencer L, Ritchie J, Lewis J, Dillon L: *Quality in qualitative evaluation: a framework for assessing research evidence.* London: Government Chief Social Researcher's Office; 2003.
32. Flemming K, Briggs M: **Electronic searching to locate qualitative research: evaluation of three strategies.** *J Adv Nurs* 2007, **57**(1):95–100.
33. Dixon-Woods M, Booth A, Sutton AJ: **Synthesizing qualitative research: a review of published reports.** *Qual Res* 2007, **7**(3):375–422.
34. Barbour R: **Checklists for improving rigour in qualitative research: a case of the tail wagging the dog?** *Br Med J* 2001, **332:**1115–1117.
35. Dixon-Woods M, Sutton A, Shaw R, Miller T, Smith J, Young B, Bonas S, Booth A, Jones D: **Appraising qualitative research for inclusion in systematic reviews: a quantitative and qualitative comparison of three methods.** *J Health Serv Res Policy* 2007, **12**(1):42–47.
36. Harden A, Gough D: **Quality and relevance appraisal**. In *An introduction to systematic reviews.* Edited by Gouhg D, Oliver S, Thomas J. London: Sage; 2012.
37. Questions to help you make sense of qualitative research; 2002. Available at http://www.casp-uk.net/wp-content/uploads/2011/11/CASP_Qualitative_Appraisal_Checklist_14oct10.pdf.
38. The Joanna Briggs Institute: *Joanna Briggs Institute Reviewers.* 2008th edition. The Joanna Briggs Institute; 2008.
39. Dixon-Woods M, Shaw RL, Agarwal S, Smith JA: **The problem of appraising qualitative research.** *Qual Saf Health Care* 2004, **13:**223–225.
40. Malterud K: **Qualitative research: standards, challenges, and guidelines.** *Lancet* 2001, **358:**483–488.
41. Popay J, Rogers A, Williams G: **Rationale and standards for the systematic review of qualitative literature in health services research.** *Qual Health Res* 1998, **8:**341–351.
42. Brunton G, Harden A, Rees R, Kavanagh J, Oliver S, Oakley A: *Children and physical activity: a systematic review of barriers and facilitators.* London: EPPI-Centre, Social Science Research Unit, Institute of Education, University of London; 2003.

doi:10.1186/1471-2288-12-181
Cite this article as: Tong *et al.*: **Enhancing transparency in reporting the synthesis of qualitative research: ENTREQ.** *BMC Medical Research Methodology* 2012 **12**:181.

Submit your next manuscript to BioMed Central and take full advantage of:

- **Convenient online submission**
- **Thorough peer review**
- **No space constraints or color figure charges**
- **Immediate publication on acceptance**
- **Inclusion in PubMed, CAS, Scopus and Google Scholar**
- **Research which is freely available for redistribution**

Submit your manuscript at
www.biomedcentral.com/submit

BioMed Central
